# The Cost of Christmas Festivities

**Published:** 1915-01-09

**Authors:** 


### THE EDITOR'S LETTER-BOX.

### The Cost of Christmas Festivities.

*To the Editor of* The Hospital.

Sir,—I notice in your account of Christmas at Addenbrooke's Hospital it is stated that the cost of the Christmas festivities is defrayed out of the fund specially raised for the purpose. I am glad of the opportunity of correcting this statement, and at the same time bringing the facts of the case to the notice of the staff, committee, and public generally. A large proportion of the cost, so far as the wards are concerned, has, at any rate in previous years, been borne by the ward sisters, who have contributed between them a sum as much as half the total raised from all other sources, and in individual cases a sum amounting to a quarter of their total yearly salary. I have not gone into the figures for the last occasion, when the ward hospitality has not been on so lavish a scale on account of the war. But there can still be no question that they will again be considerably out of pocket. I feel that it is only necessary for these facts to become known to bring about an alteration in the conditions.—I am, Sir, yours faithfully,

"Also Hit."

[The cost of Christmas festivities is an old subject of debate. We should, therefore, welcome further expressions of opinion from those concerned. We have little doubt that the facts would bear a closer scrutiny than that devoted to them by our correspondent.—Ed. The Hospital.]

